# The impact of sarcopenia and body mass index on treatment toxicity and survival in patients with extranodal natural killer/T-cell lymphoma: a retrospective analysis

**DOI:** 10.3389/fonc.2026.1765623

**Published:** 2026-07-29

**Authors:** Li Jiang, Xiaowen Yao, Yuning Deng, Pengfei Li

**Affiliations:** 1 State Key Laboratory of Oncology in South China, Collaborative Innovation Center for Cancer Medicine, Sun Yat-sen University Cancer Center, Guangzhou, China; 2 Department of VIP Region, Sun Yat-sen University Cancer Center, Guangzhou, China; 3 Collaborative Innovation Center for Cancer Medicine, Sun Yat-sen University Cancer Center, Guangzhou, China; 4 Department of Traditional Chinese Medicine, Southern Medical University Hospital of Integrated Traditional Chinese and Western Medicine, Guangzhou, China

**Keywords:** body mass index, extranodal NK/T-cell lymphoma, sarcopenia, survival, treatment toxicity

## Abstract

**Background:**

Sarcopenia and body mass index (BMI) have been associated with treatment tolerance and prognosis in various malignancies; however, their clinical significance in extranodal natural killer/T-cell lymphoma (ENKTL) remains unclear. This study evaluated the impact of sarcopenia and BMI on treatment-related toxicities and survival outcomes in ENKTL.

**Methods:**

We retrospectively analyzed 347 patients with newly diagnosed ENKTL. Sarcopenia was assessed using sex-specific skeletal muscle index thresholds at the third lumbar vertebra level. BMI was categorized as underweight, normal weight, or overweight according to Asian-specific criteria. Associations with toxicities, overall survival (OS), and progression-free survival (PFS) were evaluated using regression and survival analyses.

**Results:**

Sarcopenia was identified in 113 patients (32.6%) and was associated with adverse clinical characteristics. Compared with non-sarcopenic patients, those with sarcopenia had significantly higher risks of hematological toxicity (RR = 1.15, 95% CI 1.06–1.24, *p* = 0.001) and gastrointestinal toxicity (RR = 1.46, 95% CI 1.10–1.95, *p* = 0.009). Sarcopenia was associated with inferior OS (log-rank p = 0.004) and remained an independent predictor of mortality in multivariable analysis (aHR = 1.58, 95% CI 1.10–2.27, *p* = 0.013), but not of PFS (aHR = 1.31, 95% CI 0.93–1.84, *p* = 0.119). Underweight patients experienced the highest toxicity burden and poorest survival, whereas overweight patients showed the most favorable outcomes. Exploratory analyses suggested that patients with both sarcopenia and low BMI had the highest mortality risk.

**Conclusion:**

Sarcopenia is independently associated with poorer overall survival and increased treatment-related toxicity in ENKTL. Low BMI further identifies patients at increased risk of adverse outcomes. Incorporating body composition assessment into routine clinical evaluation may improve risk stratification and supportive care.

## Introduction

Extranodal natural killer/T-cell lymphoma (ENKTL) is a rare but highly aggressive subtype of non-Hodgkin lymphoma characterized by angioinvasion, extensive necrosis, and a strong association with Epstein–Barr virus (EBV) infection (1). The disease predominantly affects populations in East Asia and Latin America, with distinct clinical and epidemiological profiles compared with other lymphomas (2). Although advances in L-asparaginase–based chemotherapy and combined chemoradiotherapy regimens have improved outcomes for some patients, treatment-related toxicities and relapse rates remain substantial. Therefore, beyond tumor-related parameters, host factors that reflect physiological reserve and metabolic status are increasingly recognized as key contributors to the prognostic variability in ENKTL (3). Host-related factors including body composition may have particular prognostic relevance in ENKTL.

Host-related factors including nutritional and physical status have been increasingly recognized as crucial determinants of treatment tolerance and survival in malignancies (4, 5). Among these, sarcopenia—defined as a progressive loss of skeletal muscle mass and strength—has emerged as a significant biomarker of poor outcomes across multiple tumor types (6). Quantitative assessment of skeletal muscle mass using cross-sectional computed tomography (CT) at the third lumbar vertebra (L3) level provides an objective approach for identifying sarcopenia (7). Recent meta-analyses have confirmed that sarcopenia independently predicts inferior overall survival (OS), higher treatment-related toxicity, and increased postoperative complications in patients with various cancers (8). Furthermore, sarcopenia has shown particular prognostic relevance in lymphoid malignancies, where systemic inflammation and catabolic activity are pronounced (9). Emerging evidence suggests that sarcopenia may reflect an impaired host reserve that compromises chemotherapy tolerance and diminishes the capacity to recover from treatment-induced toxicity (10).

Body mass index (BMI) is another widely used anthropometric index reflecting overall nutritional and metabolic status. However, its prognostic role in oncology remains complex. Low BMI often indicates malnutrition and vulnerability, whereas higher BMI has been paradoxically associated with favorable outcomes in some malignancies, a phenomenon known as the obesity paradox (11). Importantly, BMI alone cannot distinguish between muscle and adipose tissue compartments; thus, individuals with high BMI but reduced skeletal muscle mass—termed sarcopenic obesity or sarcopenic overweight—may be misclassified as having adequate or even excessive nutritional reserves (12). Accumulating evidence suggests that sarcopenic obesity confers worse clinical outcomes than either sarcopenia or obesity alone, including heightened treatment toxicity, poorer survival, and diminished quality of life (13, 14).

Given the growing recognition of host-related factors in lymphoma prognosis, both sarcopenia and BMI have emerged as clinically relevant indicators of a patient’s physiological reserve. Sarcopenia reflects a direct loss of skeletal muscle mass and strength, whereas BMI provides a generalized estimation of nutritional and metabolic status. Although each has been independently associated with treatment tolerance and survival in various malignancies, evidence regarding their prognostic roles in ENKTL remains limited. Therefore, the present retrospective study aimed to evaluate the individual impact of sarcopenia and BMI on treatment-related toxicities, OS, and progression-free survival (PFS) in patients with ENKTL. By examining these two parameters in parallel, we sought to establish a more comprehensive framework for clinical risk assessment and supportive management in this rare but challenging lymphoma subtype.

## Methods

### Study population

**Figure 1:**
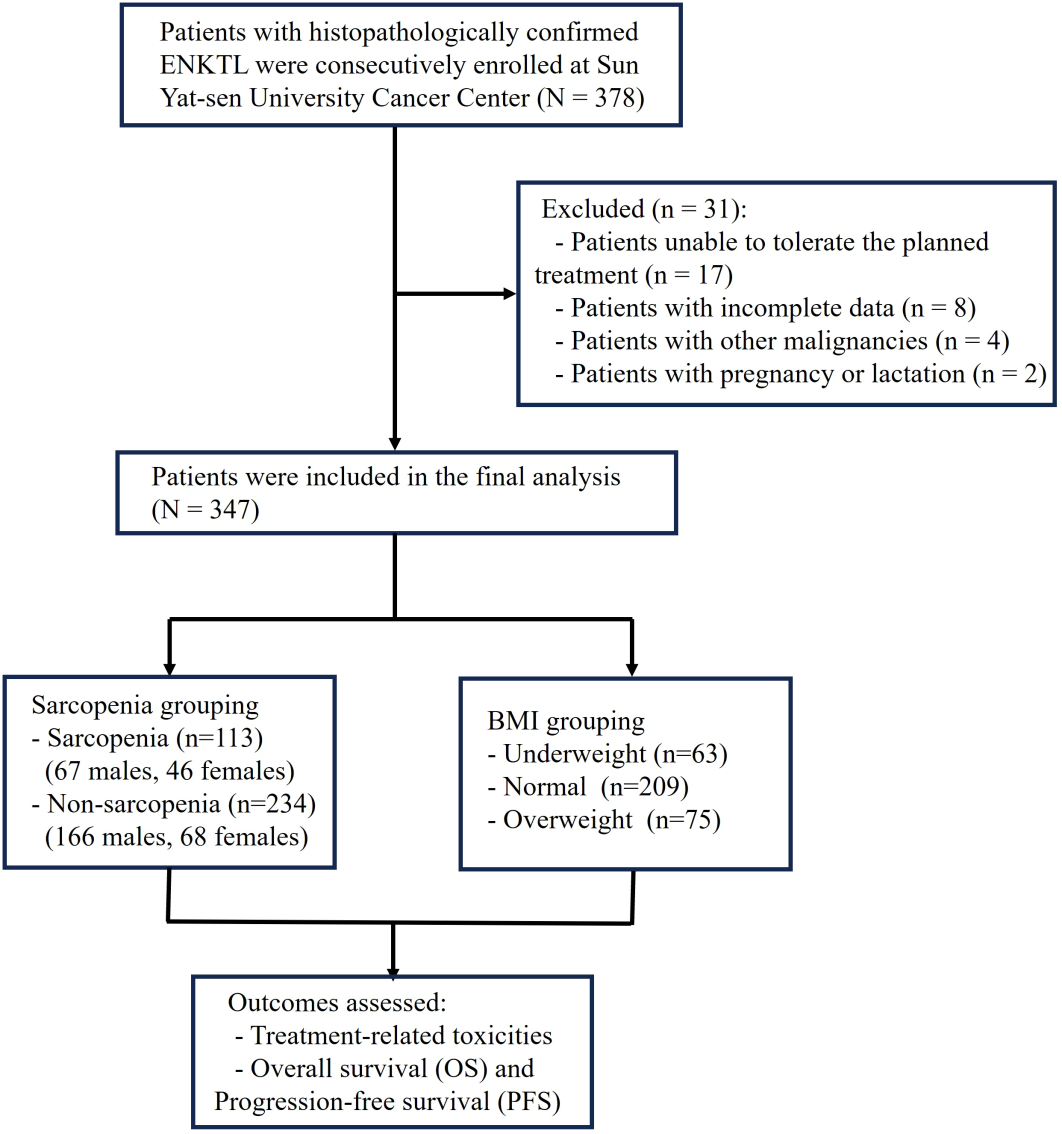
Research flowchart.

Newly-diagnosed patients with histopathologically confirmed ENKTL were consecutively enrolled at Sun Yat-sen University Cancer Center between January 2010 and December 2022.The inclusion criteria were as follows. (1) histopathologically confirmed ENKTL; (2) age >18 years; (3) availability of baseline abdominal or pelvic CT images at L3 level for body composition analysis; (4) at least one measurable lesion; (5) no prior history of other malignancies; and (6) complete clinical and follow-up data. The exclusion criteria were: (1) history of concurrent active malignancies; (2) presence of severe infection, hepatic, renal, or cardiopulmonary dysfunction, rendering them unable to tolerate the planned treatment; (3) patients with unstable vital signs nearing the end of life; (4) pregnant or lactating women; and (5) missing or incomplete clinical or follow-up data. The detailed research flowchart can be seen in **Figure 1**. This study involving human participants was approved by the Ethics Committee of Sun Yat-sen University Cancer Center, No.: 2023-45, Approval No.: B2026-119-01. The study was conducted in accordance with the Declaration of Helsinki and relevant national and institutional regulatory requirements.

### Data collection

Patient data were retrospectively collected from the hospital’s electronic medical record system. The collected baseline information included: (1) demographic characteristics, including age and sex; (2) clinical features, including primary tumor site, sites of involvement, serum lactate dehydrogenase (LDH) level, Ann Arbor stage, the presence of B symptoms (defined as unexplained fever >38 °C for more than 3 consecutive days, night sweats, or unintentional weight loss >10% within the previous 6 months), and the Eastern Cooperative Oncology Group (ECOG) performance status; (3) treatment details, specifically the chemotherapy regimens, the administration of combined radiotherapy, and the radiotherapy dose; and (4) treatment-related toxicities, which were graded according to the Common Terminology Criteria for Adverse Events (CTCAE) version 5.0. Furthermore, treatment response at various stages and survival outcomes were documented for subsequent analysis.

### Definitions and grouping

Sarcopenia was defined using pre-treatment baseline CT images. The cross-sectional area of all skeletal muscles at the level of L3 was outlined to calculate the skeletal muscle area (SMA, cm²) and the skeletal muscle index (SMI, cm²/m²). Given the fundamental sex differences in skeletal muscle mass and the potential for sex-related misclassification bias, we adopted sex-specific SMI thresholds for the primary analysis. Following the widely validated criteria proposed by Prado et al., sarcopenia was defined as SMI ≤ 52.4 cm²/m² for male patients and SMI ≤ 38.5 cm²/m² for female patients (15). Using this criterion, 113 patients (32.6%) were classified as having sarcopenia (46 females and 67 males), while 234 patients (67.4%) were categorized as non-sarcopenic (68 females and 166 males). Two radiologists independently assessed L3 skeletal muscle area. Intra- and inter-observer reproducibility was excellent (ICC = 0.92 and 0.90).

BMI was categorized into three groups according to widely accepted criteria for Asian populations: underweight (BMI < 18.5 kg/m²), normal weight (18.5 ≤ BMI < 24 kg/m²), and overweight (BMI ≥ 24 kg/m²). Based on these cutoffs, 63 patients (18.2%) were classified as underweight, 208 patients (59.9%) as normal weight, and 76 patients (21.9%) as overweight. The SMI and BMI were obtained during the baseline evaluation within two weeks before treatment initiation. This time window was selected because pre-treatment measurements best reflect the patient’s natural physiological status, while avoiding treatment-induced changes that may confound body composition assessment, which is consistent with standard practice in prior oncology studies (16).

### Study endpoints

The primary endpoint of this study was the occurrence of any and grade ≥ 3 adverse events during the treatment period. Secondary endpoints included OS and PFS. OS was defined as the time from the date of diagnosis to death from any cause. PFS was defined as the time from the date of diagnosis to the first documented disease progression, relapse, or death from any cause.

For follow-up, all patients were scheduled for clinical visits every 3 months for the first 2 years after completing treatment, and every 6 months thereafter. For patients who failed to attend scheduled clinic visits for over 6 months, follow-up was conducted via telephone. The final follow-up date for this analysis was August 30, 2025.

### Statistical analysis

Categorical variables were summarized as frequencies and percentages and compared using the chi-square test or Fisher’s exact test, while continuous variables were expressed as mean ± standard deviation (SD) or median (interquartile range, IQR) and compared using the Student’s t-test or Mann–Whitney U test, as appropriate. Treatment-related toxicities were analyzed using generalized linear models, with relative risks (RRs) and 95% confidence intervals (CIs) estimated using log-binomial regression models or Poisson regression models with robust standard errors when convergence was not achieved. OS and PFS were estimated using the Kaplan–Meier method and compared using the log-rank test. Univariate and multivariate Cox proportional hazards regression analyses were performed to identify factors associated with survival outcomes. Variables with *p* < 0.10 in univariate analysis and clinically relevant covariates identified from previous literature, including age, sex, ECOG performance status, Ann Arbor stage, serum LDH level, ENKTL subtype, B symptoms, KPI score, and treatment modality, were included in the multivariate models. Adjusted hazard ratios (aHRs) with 95% CIs were reported. All statistical analyses were performed using SPSS version 26.0 (IBM Corp., Armonk, NY, USA), and a two-sided *p* < 0.05 was considered statistically significant.

## Results

### Baseline characteristics stratified by sarcopenia status

Patients with sarcopenia showed marked differences in several clinical features compared with those without sarcopenia (**Table 1**). The sarcopenia group had a significantly different gender distribution (40.7% female vs. 59.3% male, *p* = 0.028). Patients with sarcopenia were more likely to have poorer ECOG performance status (ECOG PS ≥2: 12.4% vs. 5.6%, *p* = 0.032). Elevated serum LDH levels were more common in patients with sarcopenia. The distribution of ENKTL subtypes also differed significantly; extranasal disease occurred more frequently in the sarcopenia group. Systemic symptoms were more prevalent among patients with sarcopenia, with a higher proportion exhibiting B symptoms. Lymph node invasion was also more common in the sarcopenia cohort. In addition, a greater percentage of sarcopenic patients had a higher KPI score and a higher IPI score, indicating more aggressive disease features. No significant differences were observed between groups in terms of age distribution, Ann Arbor stage, or treatment modality (*p* > 0.05).

**Table 1:** Baseline characteristics stratified by sarcopenia status.

Variable	Category	No Sarcopenia, N (%)	Sarcopenia, N (%)	P
Gender	Female	68 (29.1%)	46 (40.7%)	0.028
	Male	166 (70.9%)	67 (59.3%)	
Age	>60 years	28 (12.0%)	19 (16.8%)	0.203
	≤60 years	206 (88.0%)	94 (83.2%)	
ECOG PS	0–1	221 (94.4%)	99 (87.6%)	0.032
	≥2	13 (5.6%)	14 (12.4%)	
Serum LDH levels	Elevated	52 (22.2%)	43 (38.1%)	0.002
	Normal	182 (77.8%)	70 (61.9%)	
Ann Arbor stage	I–II	197 (84.2%)	86 (76.1%)	0.067
	III–IV	37 (15.8%)	27 (23.9%)	
ENKTL Subtypes	Extranasal	38 (16.2%)	38 (33.6%)	0.001
	Nasal	196 (83.8%)	75 (66.4%)	
B symptoms	No	147 (62.8%)	53 (46.9%)	0.005
	Yes	87 (37.2%)	60 (53.1%)	
Lymph node invasion	No	135 (57.7%)	48 (42.5%)	0.007
	Yes	99 (42.3%)	65 (57.5%)	
IPI score	0–1	190 (81.2%)	80 (70.8%)	0.027
	≥2	44 (18.8%)	33 (29.2%)	
KPI score	0–1	158 (67.5%)	49 (43.4%)	<0.001
	≥2	76 (32.5%)	64 (56.6%)	
Treatment	Chemo + Radiotherapy	63 (26.9%)	35 (31.0%)	0.434
	Chemotherapy	171 (73.1%)	78 (69.0%)	

**Figure 2:**
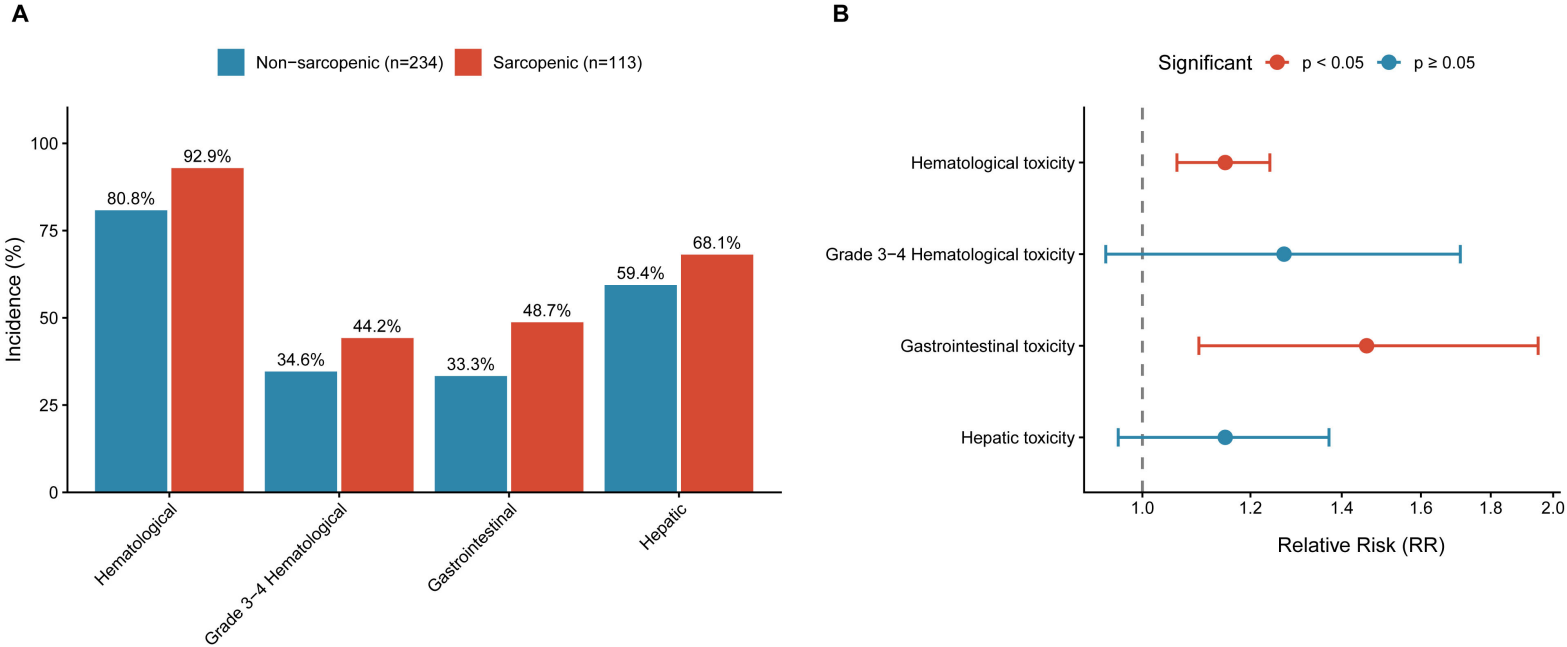
Impact of sarcopenia on treatment-related toxicities. **(a)** Comparison of treatment-related toxicities by sarcopenia status; **(b)** forest plot of risk ratios for treatment-related toxicities.

The incidence of treatment-related toxicities stratified by sarcopenia status is shown in **Table 2** and **Figure 2A**. Compared to patients without sarcopenia, those with sarcopenia had significantly higher rates of hematological toxicity, gastrointestinal toxicity, and numerically higher rates of Grade 3–4 hematological toxicity and hepatic toxicity. Among these, the differences in overall hematological toxicity (*p* = 0.001) and gastrointestinal toxicity (*p* = 0.009) were statistically significant. Risk regression analysis (**Table 2**; **Figure 2B**) indicated that sarcopenia was significantly associated with an increased risk of any-grade hematological toxicity (RR = 1.15, 95% CI 1.06–1.24, *p* = 0.001) and gastrointestinal toxicity (RR = 1.46, 95% CI 1.10–1.95, *p* = 0.009). Although the relative risks for Grade 3–4 hematological toxicity (RR = 1.27, 95% CI 0.94–1.71, *p* = 0.115) and hepatic toxicity (RR = 1.15, 95% CI 0.96–1.37, *p* = 0.128) did not reach statistical significance, a trend toward increased risk was observed.

**Table 2:** Relative risk (RR) (95% Confidence Interval) of toxicity for sarcopenia.

Toxicity	No Sarcopenia, N (%)	Sarcopenia, N (%)	RR (95% CI)	P
Hematological toxicity	189/234 (80.8%)	105/113 (92.9%)	1.15 (1.06–1.24)	0.001
Grade 3–4 Hematological toxicity	81/234 (34.6%)	50/113 (44.2%)	1.27 (0.94–1.71)	0.115
Gastrointestinal toxicity	78/234 (33.3%)	55/113 (48.7%)	1.46 (1.10–1.95)	0.009
Hepatic toxicity	139/234 (59.4%)	77/113 (68.1%)	1.15 (0.96–1.37)	0.128

### Impact of sarcopenia on survival outcomes

**Figure 3:**
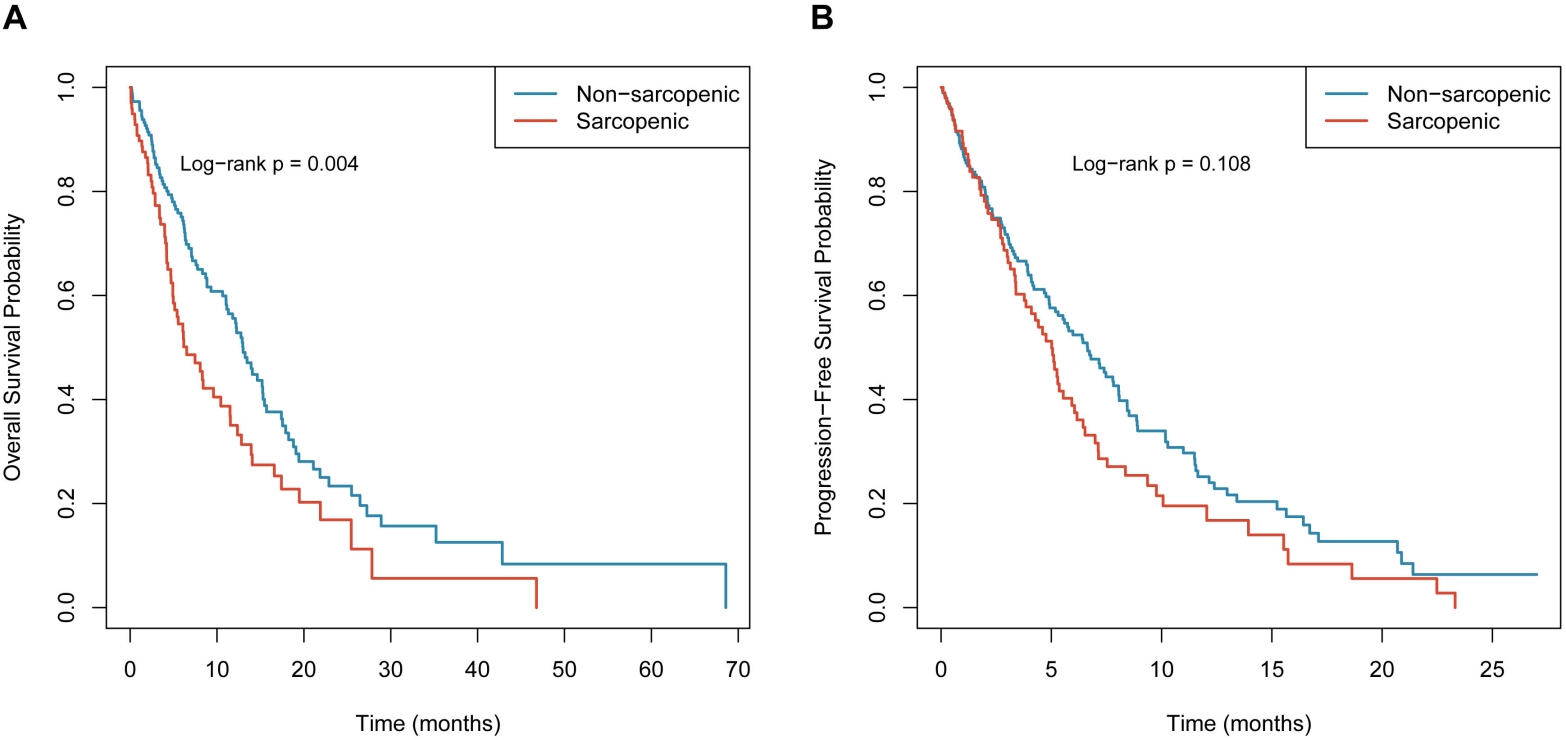
Kaplan–Meier curves for overall and PFS stratified by sarcopenia status. **(A)** Analysis of OS. **(B)** Analysis of PFS.

Kaplan–Meier survival curves for OS and PFS stratified by sarcopenia status are shown in **Figure 3**. Patients with sarcopenia exhibited significantly poorer OS compared with those without sarcopenia (log-rank *p* = 0.004). The survival curves separated early and remained consistently divergent throughout the follow-up period, indicating a substantial negative impact of sarcopenia on long-term survival. Similar to OS, the PFS curve for the sarcopenia group remained lower across the entire timeline, suggesting a potential detrimental effect, although the difference did not reach statistical significance (log-rank *p* = 0.108).

To determine whether sarcopenia independently predicts survival after adjusting for potential confounders, we performed multivariate Cox regression analysis (**Table 3**). For OS, variables with *p* < 0.10 in univariate analysis, including sarcopenia (*p* = 0.004), ECOG PS (*p* = 0.022), gender (*p* = 0.065), serum LDH (*p* = 0.072), ENKTL subtype (p = 0.015), B symptoms (*p* = 0.003), and KPI score (*p* < 0.001), were entered into the multivariate model. After adjustment for these factors, sarcopenia remained significantly associated with inferior OS, with an aHR of 1.58 (95% CI 1.10–2.27, *p* = 0.013) (**Table 3**). Other independent prognostic factors included KPI score ≥2 (aHR = 2.42, 95% CI 1.66–3.53, *p* < 0.001) and presence of B symptoms (aHR = 1.52, 95% CI 1.06–2.18, *p* = 0.022). Gender (aHR = 1.32, 95% CI 0.93–1.87, *p* = 0.118) and serum LDH (aHR = 1.29, 95% CI 0.90–1.85, *p* = 0.164) were not independently associated with OS in the multivariate model.

**Table 3:** Univariate and multivariate cox regression analysis.

Variable	OS				PFS			
	Univariate HR (95% CI)	p	Multivariate aHR (95% CI)	p	Univariate HR (95% CI)	p	Multivariate aHR (95% CI)	p
Sarcopenia (Present vs. Absent)	1.68 (1.18–2.39)	0.004	1.58 (1.10–2.27)	0.013	1.32 (0.94–1.85)	0.099	1.31 (0.93–1.84)	0.119
Age (>60 vs. ≤60 years)	1.15 (0.75–1.76)	0.523	—	—	1.07 (0.69–1.66)	0.758	—	—
Sex (Female vs. Male)	1.38 (0.98–1.94)	0.065	1.32 (0.93–1.87)	0.118	1.31 (0.93–1.85)	0.121	—	—
ECOG PS (≥2 vs. 0–1)	1.72 (1.08–2.74)	0.022	1.45 (0.90–2.34)	0.125	1.63 (1.02–2.60)	0.041	1.42 (0.88–2.29)	0.150
Ann Arbor stage (III–IV vs. I–II)	1.28 (0.88–1.86)	0.196	—	—	1.24 (0.85–1.81)	0.265	—	—
Serum LDH (Elevated vs. Normal)	1.38 (0.97–1.96)	0.072	1.29 (0.90–1.85)	0.164	1.34 (0.94–1.91)	0.104	—	—
ENKTL subtype (Extranasal vs. Nasal)	1.55 (1.09–2.20)	0.015	1.31 (0.91–1.89)	0.148	1.48 (1.04–2.12)	0.030	1.27 (0.89–1.82)	0.188
B symptoms (Present vs. Absent)	1.69 (1.19–2.40)	0.003	1.52 (1.06–2.18)	0.022	1.65 (1.16–2.35)	0.005	1.44 (1.00–2.07)	0.048
KPI score (≥2 vs. 0–1)	2.58 (1.82–3.66)	<0.001	2.42 (1.66–3.53)	<0.001	2.31 (1.62–3.30)	<0.001	2.08 (1.44–3.00)	<0.001
Treatment (Chemo alone vs. Chemo+RT)	1.10 (0.76–1.59)	0.605	—	—	1.08 (0.74–1.57)	0.695	—	—

For PFS, univariate analysis identified sarcopenia (*p* = 0.099), ECOG PS (*p* = 0.041), ENKTL subtype (*p* = 0.030), B symptoms (*p* = 0.005), and KPI score (*p* < 0.001) as candidate variables. In multivariate analysis, sarcopenia did not achieve statistical significance (aHR = 1.31, 95% CI 0.93–1.84, *p* = 0.119), while KPI score ≥2 remained an independent predictor of worse PFS (aHR = 2.08, 95% CI 1.44–3.00, *p* < 0.001) and B symptoms showed a borderline significant association (aHR = 1.44, 95% CI 1.00–2.07, *p* = 0.048).

### Baseline characteristics stratified by BMI categories

Baseline characteristics across the underweight, normal weight, and overweight BMI groups are summarized in **Table 4**. Significant differences were observed in several demographic and clinical parameters. Gender distribution varied significantly with BMI (*p* < 0.001), showing a progressive increase in the proportion of male patients across higher BMI categories (46.0% underweight, 70.8% normal weight, 84.0% overweight). ENKTL subtype also differed significantly (*p* = 0.014), with extranasal disease being more common in the underweight group (33.3%) and nasal-type disease predominating in overweight patients (89.3%). Furthermore, a consistent trend was observed for both B symptoms (*p* = 0.006) and KPI score (*p* = 0.003), with the highest prevalence in the underweight group (54.0% and 55.6%, respectively) and the lowest in the overweight group (25.3% for both), suggesting more aggressive disease features in patients with lower BMI. In contrast, no significant differences were found among groups regarding age, ECOG status, serum LDH level, Ann Arbor stage, lymph node invasion, IPI score, or treatment modality (*p* > 0.05).

**Table 4:** Baseline characteristics stratified by BMI categories.

Variable	Category	Underweight, N (%)	Normal weight, N (%)	Overweight, N (%)	p
Gender	Male	29 (46.0%)	148 (70.8%)	63 (84.0%)	<0.001
	Female	34 (54.0%)	61 (29.2%)	12 (16.0%)	
Age	≤60 years	56 (88.9%)	185 (88.5%)	63 (84.0%)	0.672
	>60 years	7 (11.1%)	24 (11.5%)	12 (16.0%)	
ECOG PS	0–1	58 (92.1%)	192 (91.9%)	73 (97.3%)	0.445
	≥2	5 (7.9%)	17 (8.1%)	2 (2.7%)	
Serum LDH	Normal	40 (63.5%)	151 (72.2%)	60 (80.0%)	0.118
	Elevated	23 (36.5%)	58 (27.8%)	15 (20.0%)	
Ann Arbor stage	I–II	50 (79.4%)	173 (82.8%)	64 (85.3%)	0.622
	III–IV	13 (20.6%)	36 (17.2%)	11 (14.7%)	
ENKTL Subtypes	Nasal	42 (66.7%)	162 (77.5%)	67 (89.3%)	0.014
	Extranasal	21 (33.3%)	47 (22.5%)	8 (10.7%)	
B symptoms	No	29 (46.0%)	113 (54.1%)	56 (74.7%)	0.006
	Yes	34 (54.0%)	96 (45.9%)	19 (25.3%)	
Lymph node invasion	No	27 (42.9%)	108 (51.7%)	46 (61.3%)	0.092
	Yes	36 (57.1%)	101 (48.3%)	29 (38.7%)	
IPI score	0–1	45 (71.4%)	165 (79.0%)	60 (80.0%)	0.368
	≥2	18 (28.6%)	44 (21.0%)	15 (20.0%)	
KPI score	0–1	28 (44.4%)	119 (57.0%)	56 (74.7%)	0.003
	≥2	35 (55.6%)	90 (43.0%)	19 (25.3%)	
Treatment	Chemoradiotherapy	42 (66.7%)	148 (70.8%)	60 (80.0%)	0.218
	Chemotherapy alone	21 (33.3%)	61 (29.2%)	15 (20.0%)	

### Impact of BMI on treatment-related toxicities

**Figure 4:**
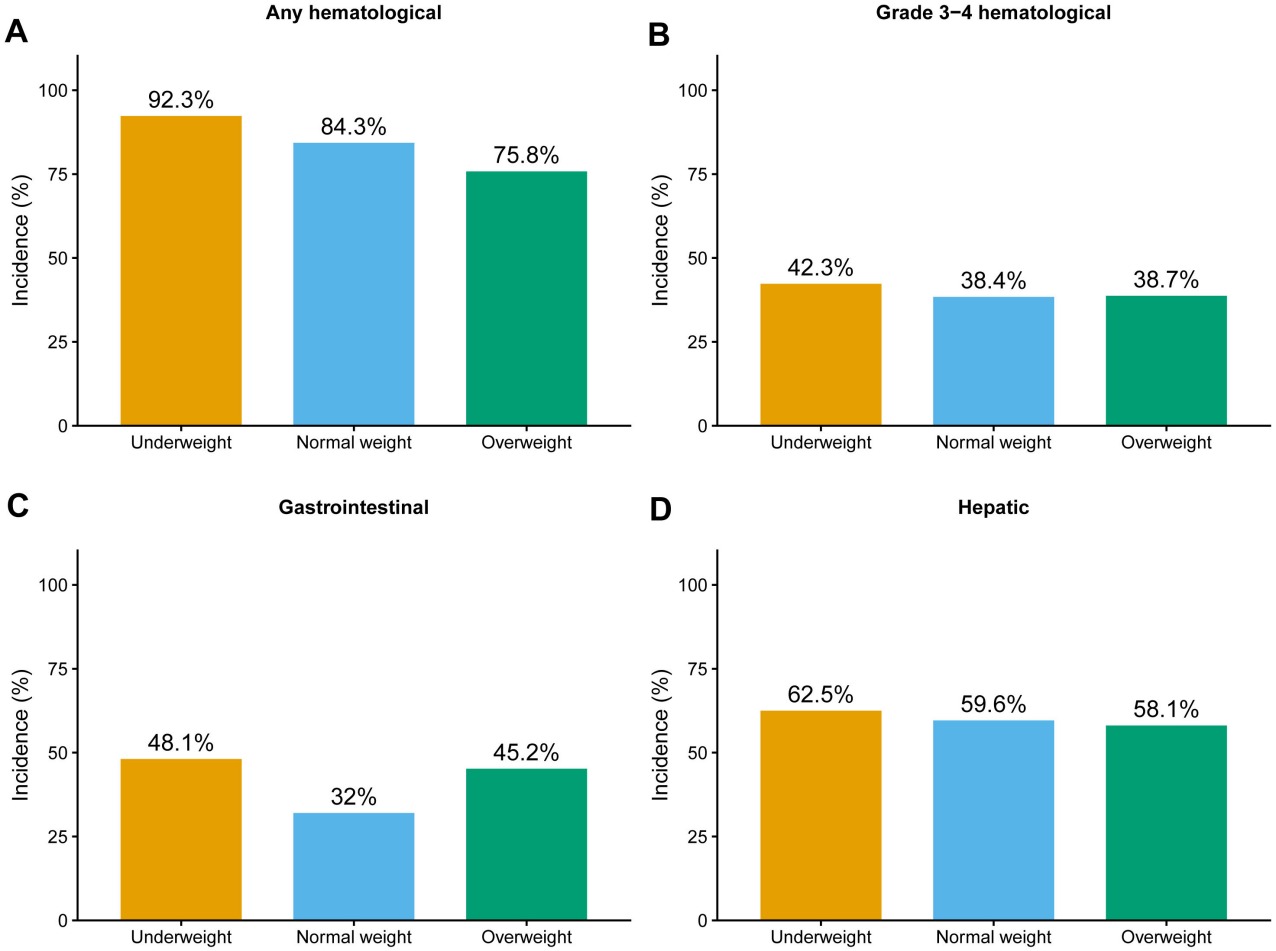
Incidence of treatment-related toxicities across BMI groups. **(A)** Overall hematological toxicity incidence. **(B)** Grade 3–4 hematological toxicity incidence. **(C)** Gastrointestinal toxicity incidence. **(D)** Hepatic toxicity incidence. (Values are presented as the number of events per total patients in each BMI category. *P* values shown in the figure are derived from the overall Pearson chi-square test comparing the three BMI groups.

The incidence of treatment-related toxicities varied among the three BMI categories. Global chi-square tests indicated significant overall differences for any hematological toxicity and gastrointestinal toxicity, but not for grade 3–4 hematological toxicity and hepatic toxicity (**Figure 4**). Specifically, underweight patients exhibited the highest incidence of any hematological toxicity (92.3%), compared to normal-weight (84.3%) and overweight patients (75.8%). Gastrointestinal toxicity was most frequent in the underweight (48.1%) and overweight groups (45.2%), with the normal-weight group showing the lowest rate (32.0%). The incidence of hepatic toxicity did not differ significantly across groups.

To further quantify the effect of BMI on treatment tolerance, log-binomial regression models were fitted using the normal-weight group as the reference. Underweight patients had a significantly increased risk of both any hematological toxicity (RR = 1.15, 95% CI 1.06–1.25, *p* = 0.001) and gastrointestinal toxicity (RR = 1.52, 95% CI 1.07–2.16, *p* = 0.019). Overweight patients demonstrated a borderline elevated risk for gastrointestinal toxicity (RR = 1.43, 95% CI 0.99–2.07, *p* = 0.056) and a borderline lower risk for any hematological toxicity (RR = 0.84, 95% CI 0.70–1.01, *p* = 0.061). No significant associations were observed between BMI and hepatic toxicity as well as Grade 3–4 hematological toxicity in any comparisons (**Table 5**).

**Table 5:** Relative risks of treatment-related toxicities across BMI groups.

Toxicity	Comparison	RR (95% CI)	p
Any hematological toxicity	Underweight vs Normal weight	1.15 (1.06–1.25)	0.001
	Overweight vs Normal weight	0.84 (0.70–1.01)	0.061
Grade 3–4 hematological toxicity	Underweight vs Normal weight	1.24 (0.86–1.79)	0.248
	Overweight vs Normal weight	1.04 (0.69–1.57)	0.842
Gastrointestinal toxicity	Underweight vs Normal weight	1.52 (1.07–2.16)	0.019
	Overweight vs Normal weight	1.43 (0.99–2.07)	0.056
Any liver toxicity	Underweight vs Normal weight	1.15 (0.93–1.42)	0.195
	Overweight vs Normal weight	1.03 (0.81–1.31)	0.812

### Impact of BMI on survival outcomes

**Figure 5:**
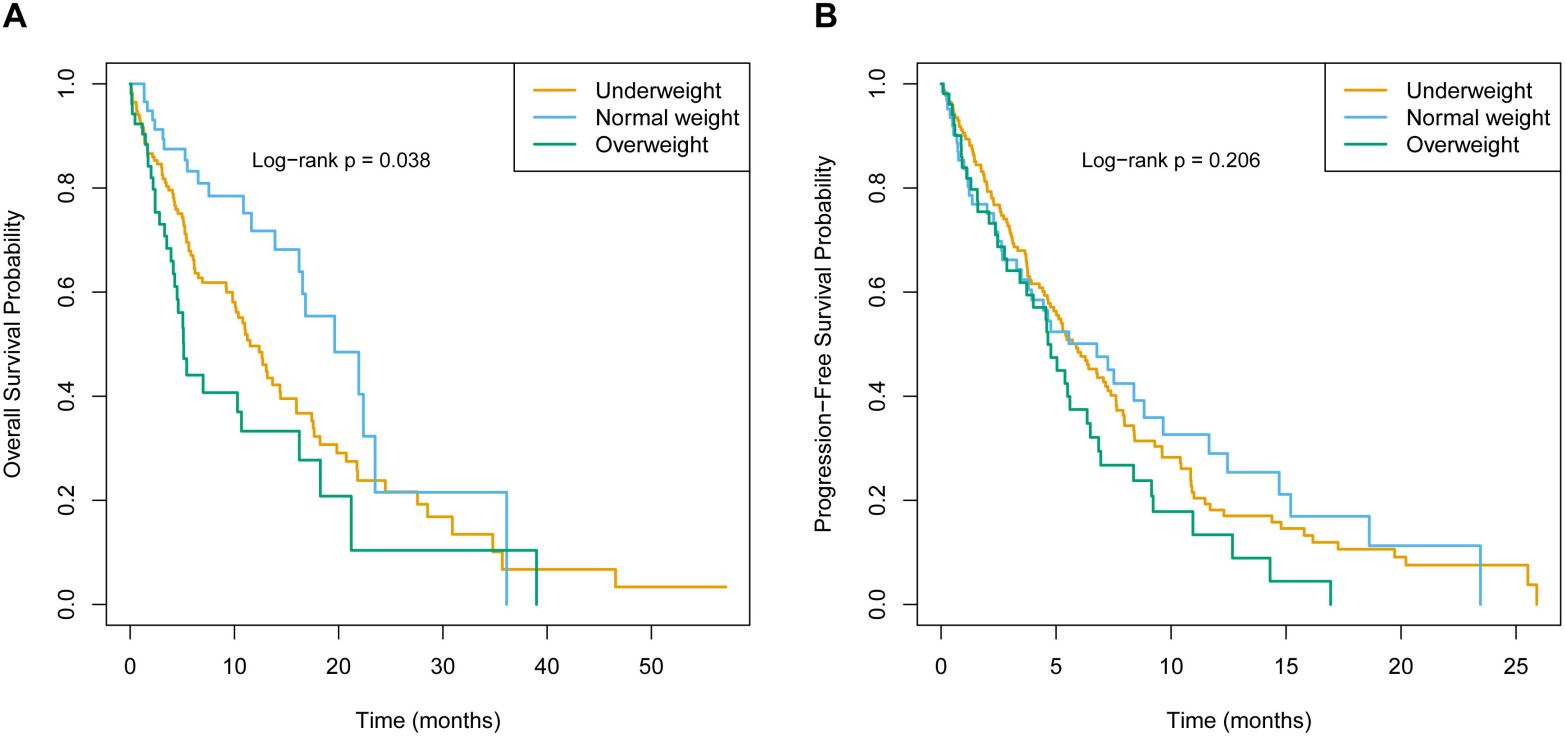
Kaplan–Meier curves for overall and PFS stratified by BMI Categories. **(A)** Analysis of OS. **(B)** Analysis of PFS.

Survival outcomes stratified by BMI categories are shown in **Figure 5**. For OS, a significant difference was observed among groups (log-rank *p* = 0.038), with the most favorable outcome in overweight patients and the poorest in underweight patients. A similar, though non-significant, trend was noted for PFS (log-rank *p* = 0.206). Collectively, these findings suggest that higher BMI is linked to improved survival, whereas being underweight portends an unfavorable prognosis in ENKTL. As shown in **Supplementary Figure 1**, mortality rates varied across the six BMI–sarcopenia subgroups, ranging from 19.6% in overweight non-sarcopenic patients to 51.9% in underweight sarcopenic patients.

## Discussion

This study confirms that sarcopenia is an important adverse prognostic factor among patients with ENKTL, consonant with prior findings in hematologic malignancies (9). Our data show that sarcopenia is significantly associated with unfavorable clinical features, including elevated LDH, more frequent B symptoms, and higher KPI scores, suggesting that sarcopenia may reflect a generalized decline in physiological reserve. Large-scale reviews have demonstrated that sarcopenia is associated with poorer survival, reduced treatment tolerance, and higher complication rates, especially among older adults (17). In cancer populations, sarcopenia has been consistently linked to increased mortality and treatment-related toxicity (18). Pre-treatment sarcopenia has been shown to predict shorter PFS among lymphoma patients undergoing autologous stem cell transplantation, indicating that muscle reserve influences treatment tolerance and disease control in intensive settings (10).

Mechanistically, tumor-associated systemic inflammation is central to muscle wasting. In EBV-associated tumors, elevated pro-inflammatory cytokines including TNF-α and IL-6 can activate the ubiquitin–proteasome pathway and muscle-specific proteolytic systems, promoting protein catabolism while suppressing muscle synthesis, perpetuating a vicious cycle between tumor inflammation and sarcopenia (19, 20). Muscle loss can also alter drug distribution and metabolism, potentially increasing effective exposure of chemotherapy drugs and exacerbating hematologic and organ toxicity (19). This is particularly relevant in ENKTL, where L-asparaginase-based regimens are widely used and have inherent hematologic toxicity; accordingly, we observed significantly higher hematological toxicity in sarcopenic patients (RR = 1.15, *p* = 0.001) *(*21). In conclusion, sarcopenia contributes to increased treatment-related toxicity and poor survival in ENKTL.

BMI also showed independent prognostic value. Underweight patients exhibited higher toxicity and poorer OS, while overweight patients had more favorable outcomes, resembling the obesity paradox reported in other malignancies. BMI is a crude indicator that cannot distinguish fat from muscle (22). Individuals with the same BMI may have very different body compositions: some overweight patients may preserve muscle mass, while others may have sarcopenic obesity, which leads to worse outcomes (23). The protective effect of higher BMI may reflect better nutritional reserves and metabolic flexibility, enabling patients to better withstand chemotherapy-induced catabolic stress (24). Adipose tissue can serve as an energy reservoir and secrete adipokines with anti-inflammatory effects (25). Conversely, underweight patients often have malnutrition with scant fat and muscle stores, impairing drug distribution and increasing toxicity risk. Adipose tissue may also modulate the immune landscape and tumor microenvironment (26). Moderate adiposity may enhance anti-tumor immunity through adipokine signaling and T-cell responses, contributing to better outcomes in overweight individuals (27, 28). BMI alone is insufficient to capture muscle depletion, as some normal-weight or overweight patients may still exhibit substantial muscle loss. Therefore, BMI should be interpreted in conjunction with direct muscle mass indicators.

Our study revealed an inconsistency between OS and PFS. Sarcopenia was significantly associated with inferior OS but not with PFS, suggesting that the survival disadvantage of sarcopenia may be driven mainly by non-progression-related factors rather than disease control itself. This discordance may also be partly attributable to the retrospective nature of our study and the limited sample size, which could have reduced statistical power to detect modest differences in PFS. Additionally, unmeasured confounding factors inherent to retrospective analyses may have differentially affected OS and PFS assessments. In our cohort, sarcopenia was consistently associated with increased treatment-related toxicity, particularly hematological and gastrointestinal toxicity. Although the excess risk for severe hematological toxicity did not reach statistical significance, the magnitude of increase was clinically meaningful. Grade ≥3 hematological toxicity, particularly febrile neutropenia, is a key determinant of dose reduction, treatment delay, and early discontinuation (29). A recent multi-institutional study in lung cancer demonstrated that sarcopenia was independently associated with a markedly increased risk of grade ≥3 toxicity, which in turn predicted worse OS (30). Severe adverse events can directly lead to mortality through febrile neutropenia, sepsis, organ failure, or prolonged hospitalization, unrelated to lymphoma progression (19). A meta-analysis of lymphoma patients receiving hematopoietic cell transplantation found that although sarcopenia increased mortality risk, there was no significant difference in non-relapse mortality between sarcopenic and non-sarcopenic groups (31), suggesting that sarcopenia-related mortality involves both disease-related and non-disease-related pathways. In the specific context of ENKTL, a study using MRI-based masticatory muscle index reported that sarcopenia independently predicted both OS and PFS (32), indicating that the strength of association may vary by assessment method, patient selection, and treatment protocol. In our cohort, reduced physiological reserve in sarcopenic patients likely weakened their ability to tolerate full-dose chemotherapy and recover from toxicity (18), leading to treatment delays or interruptions that affect long-term survival without necessarily accelerating disease progression. Sarcopenia is also associated with systemic inflammation and immune dysregulation, which may increase susceptibility to post-treatment infections (20). Our moderate sample size may have limited statistical power to detect modest differences in PFS, given ENKTL’s heterogeneity and variable responses to L-asparaginase-based regimens. Overall, sarcopenia adversely affects ENKTL outcomes primarily through reduced treatment tolerance and increased toxicity-related mortality. The OS-PFS inconsistency highlights the importance of considering non-progression-related mortality as a key component of sarcopenia-associated risk.

Multivariate Cox regression confirmed that after adjusting for gender, serum LDH, ENKTL subtype, B symptoms, KPI score, and ECOG PS, sarcopenia remained an independent predictor of inferior OS (aHR = 1.58, *p* = 0.013). This indicates that sarcopenia’s adverse prognostic effect is not merely a surrogate for other high-risk disease features but represents an independent host-related risk factor. The effect size is comparable to that reported in a recent meta-analysis of hematologic malignancies (33). The slightly lower magnitude in our study may reflect ENKTL’s unique biology, where EBV-driven inflammation may play a more prominent role than host reserve alone. These results support incorporating sarcopenia assessment into pre-treatment risk stratification for ENKTL patients (33).

Although BMI and sarcopenia overlap to some extent, they reflect distinct body composition dimensions. In our supplementary analysis, patients who were both underweight and sarcopenic had the highest mortality rate (51.9%), whereas overweight non-sarcopenic patients had the lowest (19.6%). Although limited subgroup sample sizes restrict statistical power, these findings merit attention. Underweight sarcopenic patients likely represent the most severe form of nutritional depletion and metabolic dysfunction, with markedly compromised physiological reserve (34). The coexistence of obesity and sarcopenia may exert synergistic detrimental effects. Obesity-driven chronic low-grade inflammation and metabolic dysregulation can accelerate muscle catabolism, while muscle loss may exacerbate metabolic dysfunction and limit physical activity, creating a feed-forward cycle that worsens clinical fragility (35, 36). In ENKTL, EBV-related immune activation and tumor-induced systemic inflammation may intensify these adverse host-tissue interactions, making this composite phenotype particularly detrimental (37). This combined phenotype may not only reflect coexisting low muscle mass and high adiposity but also drive high-risk clinical features including B symptoms, elevated LDH, and higher KPI scores. We emphasize that these subgroup findings remain hypothesis-generating due to limited sample size, but they provide a valuable rationale for future investigations.

Several limitations should be acknowledged. Some intersections included very few patients, limiting statistical power and preventing definitive conclusions about the sarcopenic obesity phenotype. Larger multicenter studies are needed to validate these observations. Furthermore, given the extended recruitment period (2010–2022) during which therapeutic strategies for ENKTL evolved considerably, we were unable to formally adjust for treatment era, chemotherapy type, or treatment intensity due to limited subgroup sample sizes. The retrospective design also means that important confounding factors including inflammatory biomarkers, metabolic indicators, comorbidity burden, unmeasured treatment-related variables, and nutritional interventions were not consistently adjusted. Seventeen patients were excluded because they could not tolerate planned treatment; these excluded patients likely had worse body composition profiles, and their exclusion may have introduced selection bias, potentially underestimating the true impact of sarcopenia and low BMI on outcomes. Future prospective studies should include functional indicators and metabolic analysis to better define frailty and physiological reserve in vulnerable populations. Our BMI definitions followed Asian specific criteria with overweight defined as BMI ≥24 kg/m² and obesity as BMI ≥28 kg/m², which differ from WHO global thresholds of overweight ≥25 and obesity ≥30. While appropriate for our Asian cohort, this limits direct comparability with Western studies and generalizability to non-Asian populations. Additionally, there is significant heterogeneity in obesity definitions across sarcopenic obesity research, with different studies using BMI, waist circumference, or body fat percentage, leading to variability in prevalence estimates and outcomes. Future studies incorporating multiple obesity indicators including central obesity measures will help clarify these relationships. Interventional studies are needed to determine whether nutritional support, exercise, or metabolic therapies improve outcomes in underweight or sarcopenic ENKTL patients. In the meantime, we recommend opportunistic assessment of sarcopenia using sex-specific SMI thresholds on routine baseline CT scans at no additional cost. This can be performed during standard radiologic review to identify high-risk patients before treatment. For sarcopenic or underweight patients, early nutritional support and prehabilitation exercises may be considered, though prospective validation is required.

## Data Availability

The original contributions presented in the study are included in the article/**Supplementary Material**. Further inquiries can be directed to the corresponding author.

## Supplementary material

Supplementary Figure 1 Interaction between BMI and sarcopenia on mortality in patients with ENKTL. **(A)** Bar chart demonstrating mortality rates across six BMI–sarcopenia combinations. **(B)** Interaction plot showing the mortality pattern across BMI categories stratified by sarcopenia status. **(C)** Heatmap illustrating mortality rates by BMI group and sarcopenia status. **(D)** Distribution of cases across BMI–sarcopenia subgroups.
